# Chitin Mixed in Potting Soil Alters Lettuce Growth, the Survival of Zoonotic Bacteria on the Leaves and Associated Rhizosphere Microbiology

**DOI:** 10.3389/fmicb.2016.00565

**Published:** 2016-04-21

**Authors:** Jane Debode, Caroline De Tender, Saman Soltaninejad, Cinzia Van Malderghem, Annelies Haegeman, Inge Van der Linden, Bart Cottyn, Marc Heyndrickx, Martine Maes

**Affiliations:** ^1^Plant Sciences Unit, Institute for Agricultural and Fisheries ResearchMerelbeke, Belgium; ^2^Technology and Food Science Unit, Institute for Agricultural and Fisheries ResearchMelle, Belgium; ^3^Department of Food Safety and Food Quality, Ghent UniversityGhent, Belgium; ^4^Department of Pathology, Bacteriology, and Poultry Diseases, Faculty of Veterinary Sciences, Ghent UniversityMerelbeke, Belgium; ^5^Department of Biochemistry and Microbiology, Ghent UniversityGhent, Belgium

**Keywords:** amplicon sequencing, chitin, *Escherichia coli* (EHEC), lettuce, phospholipid fatty acid (PLFA), rhizosphere, *Salmonella enterica*

## Abstract

Chitin is a promising soil amendment for improving soil quality, plant growth, and plant resilience. The objectives of this study were twofold. First, to study the effect of chitin mixed in potting soil on lettuce growth and on the survival of two zoonotic bacterial pathogens, *Escherichia coli* O157:H7 and *Salmonella enterica* on the lettuce leaves. Second, to assess the related changes in the microbial lettuce rhizosphere, using phospholipid fatty acid (PLFA) analysis and amplicon sequencing of a bacterial 16S rRNA gene fragment and the fungal ITS2. As a result of chitin addition, lettuce fresh yield weight was significantly increased. *S. enterica* survival in the lettuce phyllosphere was significantly reduced. The *E. coli* O157:H7 survival was also lowered, but not significantly. Moreover, significant changes were observed in the bacterial and fungal community of the lettuce rhizosphere. PLFA analysis showed a significant increase in fungal and bacterial biomass. Amplicon sequencing showed no increase in fungal and bacterial biodiversity, but relative abundances of the bacterial phyla *Acidobacteria, Verrucomicrobia*, *Actinobacteria*, *Bacteroidetes*, and *Proteobacteria* and the fungal phyla *Ascomycota*, *Basidiomycota*, and *Zygomycota* were significantly changed. More specifically, a more than 10-fold increase was observed for operational taxonomic units belonging to the bacterial genera *Cellvibrio*, *Pedobacter*, *Dyadobacter*, and *Streptomyces* and to the fungal genera *Lecanicillium* and *Mortierella*. These genera include several species previously reported to be involved in biocontrol, plant growth promotion, the nitrogen cycle and chitin degradation. These results enhance the understanding of the response of the rhizosphere microbiome to chitin amendment. Moreover, this is the first study to investigate the use of soil amendments to control the survival of *S. enterica* on plant leaves.

## Introduction

Utilization of organic amendments, such as chitin, is one of the most economical and practical options for improving soil and substrate quality, plant growth, and plant resilience (De Boer et al., 1999; El Hadrami et al., 2010; Sharp, 2013). Chitin is a biopolymer that is distributed among many water and soil organisms as it is a major constituent of the cell walls of fungi, the exoskeleton of arthropods and the shells of crustacean and nematode eggs. It is the second most abundant biopolymer in nature after cellulose, with an estimated natural production of 10^10^ tons per year (Jacquiod et al., 2013). Soil treatment with chitin has been shown to decrease the rate of infection of plant roots by nematodes (Sarathchandra et al., 1996; Radwan et al., 2012) and to increase disease suppressiveness against the fungal soil-borne pathogens *Verticillium dahliae* and *Rhizoctonia solani* (Cretoiu et al., 2013; Postma and Schilder, 2015). The mechanism behind this suppressiveness most often relates to a change in the microbiota in soil and rhizosphere (Cretoiu et al., 2013). Microorganisms, which are capable of hydrolyzing the chitinous cell wall of pathogenic fungi and nematodes eggs, increase their numbers and/or activities in response to the chitin added. In addition, also secondary responders to the added chitin confer overall pathogen suppression. Next to a direct effect on pathogens, changes in this rhizosphere microbiology may also affect the plant physiology and its capacity to be colonized by microorganisms, including plant and human pathogens (El Hadrami et al., 2010; Gu et al., 2013; Markland et al., 2015). Rhizosphere organisms are well-studied for their beneficial effects on plant growth and health, including nitrogen-fixing bacteria, mycorrhizal fungi, biocontrol agents, plant growth promoting rhizobacteria (PGPR), and fungi (PGPF; Berendsen et al., 2012). Studies have shown that these beneficial organisms in the rhizosphere can be increased by the utilization of chitin amendment in order to enhance plant growth and resilience to plant pathogens (Dutta and Isaac, 1979; Hallmann et al., 1999). In addition, chitin has also been shown to trigger plant immunity and acts as a pathogen-associated-molecular pattern (PAMP) triggering the plant defense against chitin-containing harmful organisms (de Jonge et al., 2010; Sharp, 2013).To date, no research has been done to investigate the indirect effect of chitin soil amendment on zoonotic bacterial human pathogens that can survive on fresh produce crops.

Authorities promote the consumption of fresh fruit and vegetables, but at the same time concerns have been raised about the food safety of leafy vegetables. Leafy vegetables, such as lettuce, are considered as high risk food, as various *Escherichia coli* O157:H7 and *Salmonella enterica* outbreaks have been related to the consumption of lettuce greenery that can carry these pathogens (Ward et al., 2002; Horby et al., 2003; Welinder-Olsson et al., 2004; Friesema et al., 2008; Nygård et al., 2008; Söderström et al., 2008). It is usually accepted that zoonotic bacterial pathogens enter the agricultural environment via animal feces. Feces may contaminate irrigation water and soil. Irrigation water is considered as the most likely key route of dispersal of zoonotic pathogens from feces to plants (Barak and Schroeder, 2012; Holvoet et al., 2014). The biology of *E. coli* and *S. enterica* on lettuce leaves under various conditions has been extensively studied (e.g., Brandl and Amundson, 2008; Oliveira et al., 2012; Van der Linden et al., 2014). A recent study showed that butterhead lettuce grown in greenhouses with a sprinkle irrigation system may present a potential health hazard when the green parts are contaminated near harvest (Van der Linden et al., 2013). Reduction in the survival of zoonotic bacterial human pathogens in the preharvest environment can help prevent spread of pathogens during post-harvest washing and packaging. A variety of direct control mechanisms such as disinfectans (including chlorine, hydrogen peroxide, organic acids, and ozons) are being used to reduce this preharvest survival, but there is a need to preserve food by natural means (Oliveira et al., 2015). Hence, bacteria isolated from the rhizosphere and leaves of leafy greens have been shown to suppress human pathogens (e.g., Markland et al., 2015; Oliveira et al., 2015) and chitin derivates have been found to have antibacterial activity against zoonotic bacterial pathogens (e.g., Jeon et al., 2014). However, no studies have investigated the indirect effect of chitin addition to the growing medium on the survival on zoonotic bacterial pathogens on the leaves. Growing media that could reduce the carrier capacity of crops for these pathogens would be an interesting strategy for sustainable control.

The objectives of this study were twofold. First, we studied the effect of chitin mixed in potting soil on lettuce growth and on the capacity of these lettuce plants to carry two zoonotic bacterial pathogens, *E. coli* O157:H7 and *S. enterica* on their leaves. Second, changes in the microbial rhizosphere of lettuce were assessed. We hypothesize that the chitin favors chitin-degrading microbiology in the soil, among which important populations of PGPR and PGPF, and the stimulation of these groups in the lettuce rhizosphere could make the plant leaves less prone to colonization by the human pathogens. To assess this colonization, we used selective platings as described by Van der Linden et al. (2013). To assess the microbial rhizosphere dynamics, two techniques were used: phospholipid fatty acid (PLFA) analysis and 16S and ITS2 rDNA amplicon sequencing. PLFA analysis is a gas chromatography-based technique widely used to monitor biodiversity in complex commodities such as soil. Specific PLFAs are markers for bacteria and fungi (Frostegård et al., 2011) and the 20 PLFA markers used in the present study discriminate six microbial groups: gram-positive bacteria, Gram-negative bacteria, bacteria (non-specific), actinomycetes (Actinomycetales), fungi and mycorrhiza (Nelissen et al., 2015). Amplicon sequencing has proven to be an efficient method to monitor changes in the relative abundance of bacterial and fungal genera or species in soil and rhizosphere (Caporaso et al., 2011; Lundberg et al., 2012).

## Materials and Methods

### Chitin Soil Amendment

Chitin flakes purified from crab shell were obtained from BioLog Hepp Gmbh (lot: 90200705). An amount of 2% (dry weight chitin/dry weight potting soil) was used in each experiment.

### Lettuce Growth

Pelletized butterhead lettuce seeds (*Lactuca sativa* L. var. *capitate* “Alexandria”) obtained from Rijk Zwaan Distribution B.V. (De Lier, The Netherlands), were germinated on moist filter papers (Whatman filters 2) in Petri dishes. The seedlings were transplanted into a 100% peat based-potting soil with a pH of 5.5–6.0 (Universal Substrate LP2B, Peltracom, Belgium) with and without 2% chitin (one seedling per 1.5 L pot) and placed in a growth chamber with conditions set at 19°C during day and 12°C at night, a relative humidity of 70–80%, and a photoperiod of 14 h. After 55 days, five plants per treatment were sampled for PLFA analysis, five plants per treatment were sampled for amplicon sequencing and seven plants per treatment were inoculated with *S. enterica* sv. Thompson RM1987N or *E. coli* O157:H7 (see below). At the end of the experiment (8 days after pathogen inoculation, see below), the lettuce heads were harvested and weighed.

### Bacterial Strains and Inoculation and Detection on Lettuce Leaves

Two bacterial strains were used: *S. enterica* sv. Thompson RM1987N and *E. coli* O157:H7 MB3885 (Van der Linden et al., 2013). Both strains were streaked from a glycerol frozen stock maintained at -70°C onto tryptone soya agar (TSA; Oxoid, Basingstoke, UK) and incubated at 37°C for 24 h. One colony was transferred to 10 mL of tryptone soya broth (TSB, Oxoid) and incubated at 37°C for 18 h while shaken at 200 rpm. Cells of each strain were washed twice by centrifugation (6000 × *g*, 15 min) in 50 mM phosphate buffered saline (PBS, pH 7.4). The optical density (OD) was measured at 595 nm using a microplate reader and concentrations were estimated based on an OD-colony-forming-unit (CFU) mL^-1^ standard curve. The appropriate amount of cells was resuspended in PBS to a concentration of 1 × 10^4^ CFU mL^-1^.

The plants were inoculated at a concentration of 10^4^ CFU ml^-1^ of PBS with a hand sprayer as described by Van der Linden et al. (2013). To count the pathogen concentrations on the lettuce leaves, individual leaves were placed in extraction bags with membrane filter (Bioreba) and weighed. PBS with 0.05% Tween 20 was added at a 1/1 (wt/vol) ratio and the samples were ground for ±15 s at maximum speed (Homex 6, Bioreba) until a homogenous mixture was obtained. Ten-fold dilutions of the resulting suspension were made in 0.1% peptone and 100 μl aliquots were spread-plated in duplicate on xylose lysine desoxycholate agar (XLD; Lab M, Bury, UK) overlaid with TSA for *S. enterica* (XLD-TAL) and on cefixime–tellurite sorbitol Mac Conkey agar (CT-SMAC; Lab M, Bury, UK) overlaid with TSA (CT-SMAC-TAL) for *E. coli* O157:H7 (Van der Linden et al., 2013). All plates were incubated at 37°C for 24 h. Three randomly chosen plants from each treatment were sampled at 4 and 8 days after inoculation (dai), while one plant per treatment was sampled at day 0 (= immediately after inoculation). From each plant, three middle-aged leaves were collected in a single extraction bag and analyzed for *E. coli* O157:H7 and *S. enterica* as described above. For mature lettuce, the 12th to 14th leaves in the head are considered as middle-aged. Leaf age is important factor influencing the survival of both pathogens on the leaves. Middle-aged leaves were selected because Van der Linden et al. (2013) found that the middle-aged leaves yielded the most consistent results for both pathogens, with the smallest standard deviations and smallest effect of environmental factors (which are difficult to control in the growth chamber). This was especially the case for *S. enterica*. The experiment was done twice for each pathogen. So, in total 6 leaves for 0 dai, 18 leaves for 4 dai, and 18 leaves for 8 dai were analyzed.

### Phospholipid Fatty Acid (PLFA) Analysis

Soil samples (approximately 50 g) were taken from five pots per treatment and stored at -20°C until freeze-dried. Total lipids were extracted from 6 g freeze-dried soil using phosphate buffer, chloroform, and methanol at a 0.9:1:2 ratio. Neutral, glycol- and phospho-lipids were separated by solid phase extraction with respectively chloroform, acetone and methanol. Phospholipids were saponified to obtain free fatty acids, which were subsequently methylated using 0.2 M methanolic KOH to form fatty acid methyl esters (FAMEs). FAMEs were analyzed with a capillary gas chromatograph-flam ionization detector (Perkin Elmer Clarus 600, Perkin Elmer, Waltham, MA, USA) with a col-elite-2560 column (100 m length × 0.25 mm ID, 0.25 μm film thickness, Perkin Elmer). The temperature program started at 75°C, followed by a heating rate of 10°C min^-1^ up to 180°C and a final heating rate of 2°C min^-1^ up to 240°C. External FAME and BAME mix (Sigma-Aldrich, St. Louis, MO, USA) were used as standard for PLFA identification and quantification. The C values were corrected using a working standard C19:0. The abundance of individual PLFAs was calculated in absolute C amounts (PLFA-C, C_x_ [nmol g^-1^]) based on the concentrations in the liquid extracts using the following formula:

$$C_{x}[nmol\,\;\;g^{-1}]=\frac{A_{x}\,\;\cdot \;\;c_{i}[\mu g]\;\cdot \;f\,\;\cdot \;1000}{A_{i}\,\;\cdot \;W[g]\;\cdot \;M[\mu g\,\;\mu mol^{-1}]}$$

Where C_x_ is the concentration of the fatty acid studied, A_x_ is the peak area of the fatty acid studied, A_i_ is the peak area of the internal standard, c_i_ is the absolute amount of internal standard in the vial [μg], f is the response factors of different PLFA compounds (peak area to concentration ratio compared to the internal standard; if not known, then = 1), W is the amount of soil [g], M is the molecular weight of the fatty acid [μg μmol^-1^]. Twenty PLFAs were selected as biomarker fatty acids for six distinct microbial groups: Gram-positive bacteria, Gram-negative bacteria, bacteria (non-specific), actinomycetes (Actinomycetales), fungi and mycorrhiza (Nelissen et al., 2015, **Table 1**).

### Rhizosphere Sampling and DNA Extraction

The lettuce rhizosphere was sampled according to Lundberg et al. (2012). Loose soil was manually removed from the roots by kneading and shaking. We followed the established definition of rhizosphere soil as extending up to 1 mm from the root surface. Subsequently, roots with the remaining soil aggregates were placed in a sterile 50 ml tube containing 25 ml phosphate buffer. Tubes were vortexed at maximum speed for 15 s, which released most of the rhizosphere soil from the roots and turned the water turbid. The turbid solution was then filtered through a 100 μm nylon mesh cell strainer to remove broken plant parts and large sediment. The turbid filtrate was centrifuged for 15 min at 3,200 *g* to form a pellet containing fine sediment and microorganisms. Most of the supernatant was removed and the pellets were stored at -20°C until DNA extraction. DNA was extracted from 250 mg of the pellet with the PowerSoil DNA isolation kit (Mo Bio, USA) according to the manufacturer’s instructions. This DNA was used for bacterial 16S (V3–V4) and fungal ITS2 rDNA amplicon sequencing as described below.

### 16S and ITS2 Amplicon Sequencing of the Rhizosphere Samples

The bacterial V3–V4 fragment of the 16S rRNA gene was selected for amplicon sequencing. Amplification of the fragment was done using the primers S-D-Bact-0341-b-S-17 and S-D-Bact-0785-a-A-21, described by Klindworth et al. (2013), extended with Illumina specific adaptors. Following PCR conditions were used: initial denaturation at 95°C for 3 min, followed by 25 cycles consisting of denaturation (95°C for 30 s), annealing (55°C for 30 s), and extension (72°C for 30 s) and a final extension step at 72°C for 5 min. To amplify the fungal rDNA-ITS2 region an adapted forward primer of fITS7b is from Ihrmark et al. (2012; GTGAATCATCRAATYTTTG) and the ITS4NGSr reverse primer (Tedersoo et al., 2014) were used, both extended with Illumina specific adaptors. The ITS2-PCR conditions were as above, except for 30 cycles with an annealing time of 1 min. A second PCR was done to attach dual indices and sequencing adaptors to both the V3–V4 as the ITS2 fragments, using the Nextera XT index kit (Illumina, San Diego, CA, USA). Same PCR conditions were used as in the first PCR, but eight cycles were used instead of 25 or 30 PCR cycles. Mastermixes for all PCRs were prepared using the Kapa HiFi Hotstart ReadyMix (Kapa Biosystems, Wilmington, MA, USA) according to the manufacturer’s instructions to a total amount of 25 μl (amplification of the bacterial and fungal fragments) and 50 μl (dual indices and sequencing adaptors attachment). Each PCR step was followed by a PCR product clean-up using the Highprep PCR reagent kit (MAGBIO, Gaithersburg, MD, USA). Final libraries were quality controlled using the Qiaxcel Advanced, with the Qiaxcel DNA High Resolution kit (QIAGEN, Germantwon, MD, USA) and concentrations were measured using the Quantus double-stranded DNA assay (Promega, Madison, WI, USA). The final barcoded libraries of each sample were diluted to 10 nM and pooled in a 2:1 range for bacteria and fungi respectively. Resulting libraries were sequenced using Illumina MiSeq v3 technology (2 bp × 300 bp, paired-end) by Macrogen, South-Korea.

Additionally, two technical replicates for each treatment (one control and one chitin rhizosphere, so four samples in total) were done to study the reproducibility of sequencing, with a separate DNA extraction and sequencing done on the same rhizosphere of a single plant.

### Sequence Reads Processing

Demultiplexing of the amplicon dataset was done by the sequencing provider. The raw sequence data is available in NCBI’s Sequence Read Archive under the accession number PRJNA294362. Trimmomatic v0.32 was used for removing the primers (Bolger et al., 2014). Raw Illumina forward and reverse reads were merged using the program PEAR v.0.9.8 (Zhang et al., 2014). Length cut-off values for the merged sequences were set between 400 and 450 bp for the V3–V4 and between 200 and 480 bp for the ITS2. A minimum overlap size of 120 bp and quality score threshold of 30 were used for all sequences. An extra program ITSx v.1.0.11 was used to extract the ITS2 sequences (Bengtsson-Palme et al., 2013). In the following steps, different programs of the Usearch software v7.0.1090 were used (Edgar, 2014). Merged sequences were quality filtered with a maximum expected error of 3 with the “fastq_filter” option. Next, sequences of all samples that needed to be compared to each other were merged into one file, dereplicated and sorted by abundance. Clustering the reads into operational taxonomic units (OTUs) was done using Uparse, with an identity level of 97% for V3–V4 and 98.5% for ITS2 (Ihrmark et al., 2012; Edgar, 2014). In the case of V3–V4, chimeras were removed using Uchime with the RDP Gold database as a reference (Edgar et al., 2011). Finally, sequences of individual samples were mapped back to the representative OTUs using the “usearch_global” algorithm at 97% identity, and converted to an OTU table (McDonald et al., 2012).

### Statistical Analysis and Downstream Processing of OTU Tables

Lettuce growth, zoonotic pathogens enumeration and absolute PLFA concentrations were analyzed with Statistica 12 (Statsoft) using a multi-factor analysis of variance with *P* < 0.05. Full factorial design was performed first. If all interaction terms were not significant, a *t*-test was done to compare the mean of the chitin treatment with the control treatment. For the lettuce growth, chitin (with or without) and experiment (1 and 2) were the factors with fresh weight per plant as dependent variable. For the enumeration of the zoonotic pathogens on lettuce leaves, chitin (with or without), sampling time (days 4 and 8) and experiment (1 and 2) were the factors with cfu g^-1^ lettuce leaf as dependent variable. Statistical differences in the absolute values of the PLFA’s between the different treatments were determined using a MANOVA analysis.

Statistical differences of the relative abundances in PLFA were determined using ANOVA by the Statistical Analysis of Metagenomic Profiles (STAMP) program (Parks and Beiko, 2010). Correction of multiple testing was done using the Benjamini–Hochberg False Discovery Rate method. Principal coordinate analysis, in which the dissimilarity matrices were based on the Bray–Curtis index (PCoA), on the PLFA data was done using the vegan package in R (version 2.0-10; Oksanen et al., 2010) with dissimilarity matrices calculated using the Bray–Curtis index.

Operational taxonomic units tables of the V3–V4 and ITS2 amplicon sequencing were analyzed using the QIIME software package (v1.9.0; Caporaso et al., 2010b). Representative bacterial OTU sequences were aligned to the SILVA v119 97% core set (version 119) using QIIME (Caporaso et al., 2010a; Quest et al., 2012). Taxonomy assignment was done using the uclust assignment method, accepting maximum 3 hits for each query sequence and then assigning the most specific taxonomic label that is associated with at least 51% of the hits. Similarly, taxon assignments of fungal OTU sequences were done using the UNITE database (version 7.0; Kõljalg et al., 2013).

Rarefaction analysis was done using the “alpha_rarefaction.py” script of QIIME. Rarefaction curves were estimated for both bacterial as fungal OTUs (**Supplementary Figures S5** and **S7**, respectively). Convergence was reached at 50,000 sequences for the bacterial OTUs and at 10,000 sequences for the fungal OTUs. Those rarefaction depths were used to determine the number of observed OTUs representing the bacterial and fungal richness. Shannon–Wiener diversity indices were calculated using the “alpha_diversity.py” script (QIIME) and used to estimate the within sample diversity. To find significant differences among mean richness and diversity indices, ANOVA analysis was done. Tukey HSD test was used to find the mean richness and diversity indices that are significantly different from each other. Both analysis were done using the R program (version 3.1.0; R Core Team, 2015).

Multivariate analysis was done using the specific R package vegan (version 2.0-10; Oksanen et al., 2010). Dissimilarity matrices (based on the Bray–Curtis dissimilarity index) were calculated from the OTU tables of the fungal and bacterial sequences. The OTU tables were normalized by removing those OTUs with an abundance lower than 0.01% in at least one sample. Effect of chitin addition on the bacterial and fungal communities was studied by doing a PERMANOVA analysis on these dissimilarity indices. To visualize the observed differences in bacterial community composition, PCoA on the dissimilarity matrices was done.

The STAMP analysis software was used to study individual differences in the bacterial groups (Parks and Beiko, 2010). For each experiment, ANOVA analyses were done on a species table to determine the effect of chitin addition on the individual groups (phyla, species). To correct for multiple testing, we used the Benjamini–Hochberg False Discovery Rate method. The used species table was calculated by the QIIME software (“summarize_taxa_through_plots.py”) and normalized by only keeping those species which were present with a minimal abundance of 0.01% in minimum one sample.

## Results

### Effect of Chitin Soil Amendment on Lettuce Growth and Survival of Zoonotic Pathogens on the Leaves

For the lettuce growth, there was no interaction between the treatments (with chitin and without chitin) and the two independent experiments, so data were pooled. Addition of chitin significantly (*P* = 0.003) increased the fresh weight of the lettuce plants to 213.00 ± 18.76 g per plant, compared with 172.08 ± 17.75 g per plant in the control (**Supplementary Figure S1**; left = control treatment, right = chitin treatment). Moreover, the plants in the chitin treatment showed more root development as compared to the control (**Supplementary Figure S2**).

In the control without chitin, the dynamics of *E. coli* O157:H7 concentrations on the leaves were highly similar to those reported by Van der Linden et al. (2013) who grew lettuce plants in the same conditions and using the same *E. coli* isolate. There was no interaction between the two treatments (with and without chitin), the two independent experiments and the three sampling days (0, 4, and 8 dai). However, there was an interaction between the sampling days and the two experiments, so each day was analyzed separately. For day 4, there was no interaction effect between the treatments and experiments, so data could be pooled. On day 4, there was no significant effect of the chitin on the survival of *E. coli* O157:H7. On day 8 a significant reduction of *E. coli* survival in Experiment 2 (*P* = 0.009), but not in Experiment 1 was observed (interaction effect treatment-experiment; **Supplementary Figure S3**).

**FIGURE 1:**
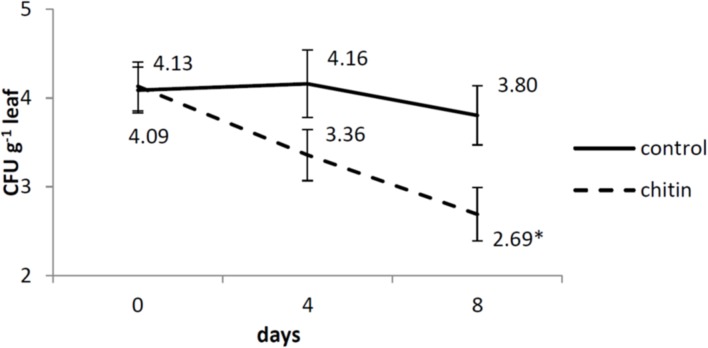
***Salmonella enterica* sv. Thompson RM1987N dynamics on middle-aged lettuce leaves at 0, 4, and 8 days after spray inoculation analyzed by plating as described by Van der Linden et al. (2013)**. Full lines represent control plants, while dashed lines represent chitin treated plants. The data are calculated from the log-transformed values of the pathogen per gram tissue from two independent experiments (*n* = 2 plants or 6 leaves for day 0 and *n* = 6 plants or 18 leaves for day 4 and 8). Asterisk means significantly different between the chitin and the control treatment. Bars represent standard errors.

Also for the dynamics of *S. enterica* on the leaves in our control, we reported highly similar results as the ones obtained by Van der Linden et al. (2013). There was no interaction between the two treatments (chitin and without chitin), the two independent experiments and the three sampling days (0, 4, 8 dai). Also no interaction was observed between the treatments and experiments, so data were pooled over the two experiments. There was an interaction between the treatments and the sampling days, so each day was analyzed separately. At day 4, no significant difference between the two treatments was found, whereas at day 8, there was significantly less survival of *S. enterica* on the leaves in the chitin treatment as compared to the control (**Figure 1**).

### Effect of Chitin Soil Amendment on Lettuce Rhizosphere Microbiology Analyzed with PLFA

The soil from five individual pots of the control treatment (= without chitin) and from five individual pots of the chitin treatment were analyzed using PLFA. Both the absolute (nmol g^-1^ dry soil) and the relative abundance (%) of each biomarker were assessed per treatment. All individual PLFA biomarkers and all microbial groups were significantly increased after chitin amendment (absolute abundances), resulting in a double amount of total biomass as compared to the control (**Table 1A**). For the relative abundance, 13 of the 20 biomarkers were significantly different from the control, with a significant decrease in relative abundance for bacteria (non-specific) and Gram positive bacteria and a significant increase for the Gram negative bacteria (**Table 1B**).

**Table 1A:** Absolute concentrations (nmol g^-1^ dry soil) ± standard error of PLFA biomarkers specific for different microbial groups in potting soil with and without 2% chitin, after 55 days of lettuce cultivation in the growth chamber.

Microbial group	PLFA biomarker	Treatment	
		Control	2% chitin
Gram positive bacteria	i-C15:0	20.22 ± 0.43	31.39 ± 2.22*
	a-C15:0	12.71 ± 0.35	19.27 ± 1.49*
	i-C16:0	7.13 ± 0.23	11.19 ± 0.77*
	i-C17:0	8.08 ± 0.20	14.91 ± 1.11*
Actinomycetales	10Me-C16:0	4.20 ± 0.20	5.83 ± 0.34*
	10Me-C17:0	4.96 ± 0.19	8.74 ± 0.56*
	10Me-C18:0	0.50 ± 0.03	3.34 ± 1.07*
			
Bacteria (non-specific)	C14:0	2.72 ± 0.10	3.70 ± 0.28*
	C15:0	2.05 ± 0.05	3.34 ± 0.23*
	C16:0	41.41 ± 1.64	71.72 ± 5.56*
	C17:0	1.17 ± 0.04	2.27 ± 0.16*
	C18:0	9.77 ± 0.26	16.36 ± 0.94*
			
Gram negative bacteria	C16:1c9	11.14 ± 0.74	25.18 ± 2.72*
	C16:1t9	4.15 ± 0.63	8.98 ± 0.65*
	C17:0cy	9.48 ± 0.53	22.56 ± 2.27*
	C18:1c11	9.77 ± 0.26	16.36 ± 0.94*
	C19:0cy	23.56 ± 0.78	49.23 ± 4.32*
			
Fungi	C18:1c9	15.26 ± 0.68	38.37 ± 4.62*
	C18:2n9,12	22.32 ± 3.28	31.50 ± 1.69*
			
Arbuscular mycorrhiza	C16:1c11	4.22 ± 0.37	8.17 ± 0.54*

Total biomass		221.82 ± 8.28	402.20 ± 29.38*

*Asterisks indicate a significant increase as compared to the control (*P* < 0.05) by analysis of variance with *n* = 5*.

**Table 1B:** Relative abundance (%) ± standard error of biomarker PLFAs and PLFA groups in potting soil with and without 2% chitin after 55 days of lettuce cultivation in the growth chamber.

Microbial group	PLFA biomarker	Treatment	
		Control	2% chitin
Gram positive bacteria	i-C15:0	9.12 ± 0.19	7.77 ± 0.27*
	a-C15:0	5.73 ± 0.17	4.76 ± 0.11*
	i-C16:0	3.21 ± 0.06	2.78 ± 0.13*
	i-C 17:0	3.64 ± 0.08	3.68 ± 0.05
Actinomycetales	10Me-C16:0	1.89 ± 0.05	1.45 ± 0.04*
	10Me-C17:0	2.23 ± 0.04	2.16 ± 0.06
	**10Me-C18:0**	0.22 ± 0.02	0.82 ± 0.25*
			
Non-specific bacteria	C14:0	1.22 ± 0.05	0.91 ± 0.02*
	C15:0	0.93 ± 0.03	0.83 ± 0.02*
	C16:0	18.6 ± 0.21	17.66 ± 0.12*
	C17:0	0.53 ± 0.02	0.56 ± 0.02
	C18:0	4.40 ± 0.07	4.05 ± 0.09*
			
**Gram negative bacteria**	**C16:1c9**	5.00 ± 0.21	6.15 ± 0.23*
	C16:1t9	1.85 ± 0.24	2.22 ± 0.07
	**C17:0cy**	4.26 ± 0.15	5.53 ± 0.17*
	C18:1c11	7.77 ± 0.23	7.30 ± 0.27
	**C19:0cy**	10.61 ± 0.24	12.11 ± 0.39*
			
	10Me-C16:0	1.89 ± 0.05	1.45 ± 0.04*
	10Me-C17:0	2.23 ± 0.04	2.16 ± 0.06
	**10Me-C18:0**	0.22 ± 0.02	0.82 ± 0.25*
Fungi	C18:1c9	6.88 ± 0.27	9.39 ± 0.66*
	C18:2n9,12	9.95 ± 1.32	7.83 ± 0.35
Arbuscular mycorrhiza	C16:1c11	1.91 ± 0.19	2.02 ± 0.08

*Asterisk indicates a significant difference to the control (*P* < 0.05) by analysis of variance with *n* = 5. Microbial groups and biomarkers marked **in bold** are significantly more abundant in the chitin treatment as compared to the control. Underlined microbial groups and biomarkers are significant less abundant in the chitin treatment as compared to the control*.

To illustrate these dissimilarities in the microbial communities of the chitin supplemented soil and the control a PCoA on the PLFA data was done (**Supplementary Figure S4**). The first principal coordinate (PCo1), which represents the major variance of the dataset (94.9%) confirmed that the microbiome differed between soil with and without chitin. The second principal coordinate describes the variation between the samples in each treatment (with and without chitin) This is only a minor source of variability (2.5%), indicating a high reproducibility of the data of the five pots per treatment.

### Effect of Chitin Soil Amendment on Bacterial Lettuce Rhizosphere Using 16S rDNA Based Amplicon Sequencing

The bacterial microbiomes present in rhizospheres of plants grown in potting soil with and without chitin were compared by sequencing the V3–V4 region of the 16S rDNA. The rhizospheres of five individual plants from each treatment (with and without chitin) were prepared and analyzed separately. After merging of the forward and reverse reads and quality filtering, 83.8% of the sequences were retained, resulting in an average of 92,549 sequences per sample. Rarefaction depth was reached at approximately 50,000 sequences, indicating that enough sequence reads were generated (**Supplementary Figure S5**). No differences were observed between the two technical replicates per treatment, indicating reproducibility of the sequencing. There were no significant differences in the number of observed OTUs and Shannon–Wiener diversity indices between the control and the chitin treatment (1436 ± 35 vs. 1370 ± 12 and 8.15 ± 0.03 vs. 8.17 ± 0.08, respectively), indicating that the chitin amendment did not increase the bacterial biodiversity in the rhizosphere. However, significant shifts in bacterial composition (taxonomic groups) were observed between the two treatments (PERMANOVA, *P* = 0.011) which is illustrated by a PCoA plot (**Supplementary Figure S6**). The first principal coordinate contains the major source of variability (51.8%) and refers to the different rhizospheres of the plants grown in chitin vs. non-chitin amended soil. The second principal coordinate describes the variation within the treatments. This is only a minor source of variability (17.8%), indicating a high reproducibility.

**FIGURE 2:**
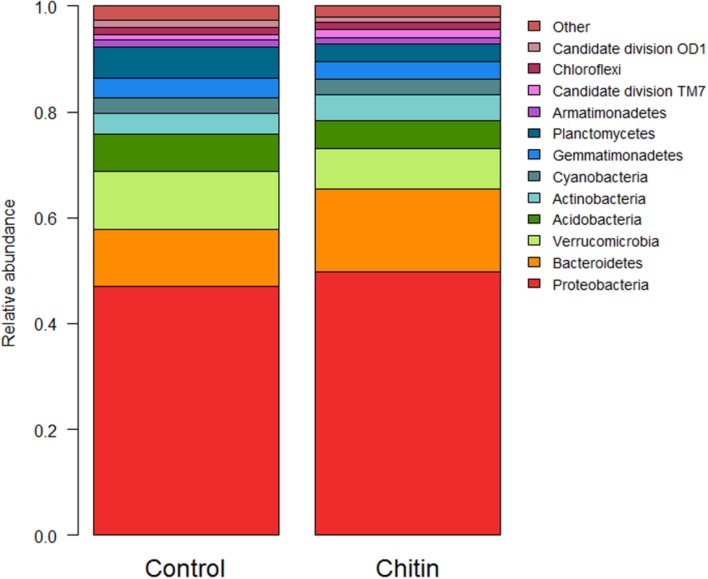
**Analysis of the bacterial composition of the lettuce rhizosphere in unamended and chitin amended potting soil**. Relative abundance (percentages) of the different bacterial phyla (16S V3–V4 region) in the lettuce rhizosphere. Phyla representing less than 1% of the total community are bundled in the group “other,” as their taxonomic composition may be uncertain.

In total, 28 bacterial phyla were found across all samples. Thirteen of these phyla showed a significant difference between the control and the chitin treatment, of which 10 phyla and 2 candidate divisions each represented more than 1% of the community (**Figure 2**). Most importantly, the relative abundances of the *Acidobacteria* and the *Verrucomicrobia* were significantly decreased in the chitin treatment as compared to the control, whereas the relative abundance of the *Actinobacteria*, *Bacteroidetes*, and the *Proteobacteria* was significantly increased in the chitin treatment as compared to the control (**Table 2**). Analyzing these five phyla together, it was shown that the relative abundance of the Gram negative bacteria was significantly increased in the chitin treatment. In contrast, the relative abundance of the Gram positive bacteria was not significantly different from the control.

**Table 2:** Relative abundance (% of sequences) ± standard error of the five most dominant bacterial phyla in the lettuce rhizosphere grown for 55 days in the growth chamber in potting soil with and without 2% chitin after.

	Treatment	
	Control	2% chitin
**Proteobacteria**	47.04 ± 0.18	49.79 ± 0.36*
**Bacteroidetes**	10.78 ± 0.12	15.54 ± 0.19*
Verrucomicrobia	10.85 ± 0.20	7.71 ± 0.25*
Acidobacteria	7.11 ± 0.13	5.37 ± 0.13*
**Actinobacteria**	3.87 ± 0.04	4.82 ± 0.08*
**Gram negative bacteria**	68.67	73.04^∗^
Gram positive bacteria	10.98	10.19
**Total**	79.65	83.24^∗^

*Asterisk indicates a significant difference to the control (*P* < 0.05) by analysis of variance with *n* = 5. The phyla **in bold** have a significantly higher relative abundance in the chitin treatment as compared to the control, the underlined phyla have significantly lower relative abundance*.

**FIGURE 3:**
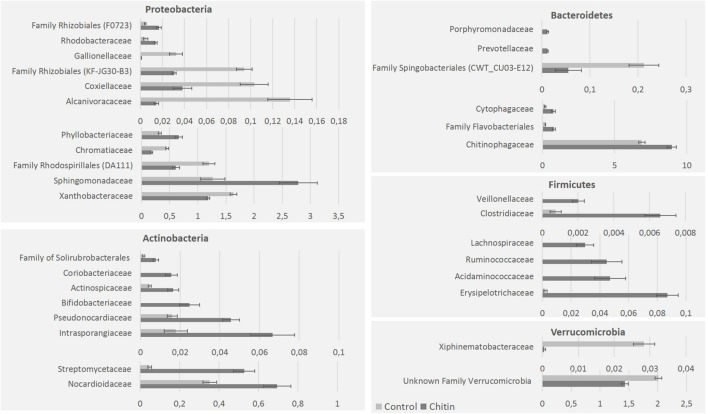
**Major bacterial taxonomical changes in the rhizosphere community after 2% chitin amendment to the potting soil**. The graphics represent the significant differences (percentages) of representative families belonging to five major bacterial phyla in the lettuce rhizosphere due to the addition of chitin to the potting soil.

On family level, the highest relative abundance was for the *Chitinophagaceae* and *Sphingomonadaceae*. Chitin altered the relative abundance of 40 bacterial families, in particular 11 families of the *Proteobacteria*, 8 of the *Actinobacteria*, 6 of the *Bacteroidetes*, 6 of the *Firmicutes*, 2 of the *Verrucomicrobia*, 1 of the *Acidobacteria* (unknown family of subgroup 6) and 1 of the phylum *Chlamydiae* (*Simkaniaceae*; **Figure 3**). Next to these families, which belonged to significantly altered phyla by chitin addition, two families of the *Chloroflexi* (*Anaerolineaceae* and an unknown family of the *Thermomicrobia*), two unknown families of the *Planctomycetes* and the *Spirochaetaceae* (Phylum: *Spirochaetes*) were significantly changed in relative abundance due to chitin addition (data not shown).

The relative abundance of 38 bacterial genera was significantly different between the rhizospheres of the two treatments, 18 genera represented more than 0.05% of the OTUs in one of the two treatments. These 18 genera are reported in **Table 3A**. Thirteen genera were significantly increased in the chitin treatment, including genera containing species that are reported to be involved in plant growth promotion, chitin degradation and biological control. Five genera, *Pseudolabrys*, *Alcanivorax*, *Candidatus solibacter*, *Nitrosococcus*, and *Aquicella* were significantly decreased.

**Table 3A:** Significant differences in the relative abundance of bacterial genera (%) ± standard error between lettuce rhizospheres in potting soil with and without 2% chitin (*n* = 5) and the possible functions of species belonging to this genera reported in literature.

Phylum	Family	Genus	Treatment		Increase or decrease	Possible functions (reference)
			Control	2% chitin		
*Proteobacteria*	***Pseudomonadaceae***	***Cellvibrio***	0.09 ± 0.05	1.34 ± 0.26	**15x**	PGP, chitin degradation and N-cycle (Kolton et al., 2011; Anderson and Habiger, 2012; Suarez et al., 2014)
	***Sphingomonadaceae***	***Sphingomonas***	0.45 ± 0.06	1.02 ± 0.07	**2x**	PGP, chitin degradation and biocontrol (Zhu et al., 2007; Wachowska et al., 2013; van Bruggen et al., 2014)
	***Sphingobacteriaceae***	***Pedobacter***	0.02 ± 0.01	0.38 ± 0.09	**19x**	PGP and biocontrol (De Boer et al., 2007)
	***Rhodospirillaceae***	***Azospirillum***	0.03 ± 0.01	0.19 ± 0.04	**6x**	PGP and N-cycle (Saharan and Nehra, 2011)
		***Dongia***	0.72 ± 0.05	1.29 ± 0.06	**2x**	*/*
	***Phyllobacteriaceae***	***Nitratireductor***	0.16 ± 0.02	0.42 ± 0.05	**3x**	N-cycle (Penton et al., 2013)
	***Bradyrhizobiaceae***	***Afipia***	0.38 ± 0.02	0.58 ± 0.04	**2x**	*/*
	*Coxiellaceae*****	*Aquicella*	0.10 ± 0.01	0.04 ± 0.01	0.4x	/
	*Xanthobacteraceae*	*Pseudolabrys*	1.53 ± 0.07	1.13 ± 0.03	0.7x	/
	*Alcanivoracaceae*	*Alcanivorax*	0.14 ± 0.02	0.01 ± 0.00	0.1x	*/*
	*Chromatiaceae*	*Nitrosococcus*	0.46 ± 0.03	0.18 ± 0.02	0.4x	N-cycle (Juretschko et al., 1998)
*Bacteroidetes*	***Cytophagaceae***	***Dyadobacter***	0.02 ± 0.0	0.33 ± 0.07	**16x**	*/*
	***Chitinophagaceae***	***Taibaiella***	0.30 ± 0.07	2.14 ± 0.42	**7x**	N-cycle (Zhang et al., 2013)
*Nitrospirae*	***Nitrospiraceae***	***Nitrospira***	0.24 ± 0.05	0.90 ± 0.10	**4x**	N-cycle (Kox and Jetten, 2015)
*Actinobacteria*	***Streptomycetaceae***	***Streptomyces***	0.05 ± 0.01	0.53 ± 0.06	**10x**	PGP, chitin degradation and biocontrol (Hjort et al., 2010; Saharan and Nehra, 2011)
	***Nocardioidaceae***	***Nocardioides***	0.11 ± 0.02	0.28 ± 0.04	**3x**	Biocontrol (Carrer et al., 2008)
*Firmicutes*	***Anaeroplasmataceae***	***Asteroleplasma***	0.00 ± 0.00	0.08 ± 0.01	–	*/*
*Acidobacteria*	*Solibacteraceae*	*Candidatus*	0.52 ± 0.03	0.17 ± 0.01	0.3x	*/*

*PGP, Plant growth promotion. **Bold** means a significantly higher relative abundance in the chitin treatment as compared to the control. Underlined means a significant decrease in the relative abundance in the chitin treatment as compared to the control*.

### Effect of Chitin Soil Amendment on the Fungal Lettuce Rhizosphere Using ITS2 Amplicon Sequencing

The fungal microbiomes present in rhizospheres of five plants that were grown in soil with or without chitin (10 rhizospheres of individual plants in total) were compared by ITS2 sequencing. After merging of the forward and reverse reads and quality filtering, 83.6% of the sequences were retained, resulting in an average of 50,045 sequences per sample. Rarefaction depth was reached at approximately 10,000 sequences (**Supplementary Figure S7**), indicating that enough sequence reads were generated. In total, around 21% of the sequences of the control and 17% of the sequences of the chitin amendment could not be assigned to a fungal phylum.

There were no significant differences in number of observed OTUs and Shannon–Wiener diversity indices between the control and the chitin amendment (298 ± 15 vs. 271 ± 11 and 4.81 ± 0.40 vs. 4.65 ± 0.10, respectively), indicating that the chitin treatment did not increase the fungal biodiversity. However, significant shifts in fungal composition (taxonomic groups) between the two treatments were observed (PERMANOVA, *P* = 0.008), illustrated by the OTU PCoA plot (**Supplementary Figure S8**). The first principal coordinate contains the major source of variability (64.8%) and reveals that the fungal rhizospheres of the chitin-grown plants are significantly different from the control plants. The second principal coordinate (18.8%) is highly reduced in the chitin treatment as compared to the control treatment. The fungal rhizosphere populations that developed in association with the plants grown in this chitin-amended soil cluster very tightly. It indicates that the chitin directs the fungal composition in a focused and consistent way and this is probably due to an high increase of the *Morteriella* species in the chitin treatment as compared to the control treatment (58.1% vs. 3.2%, **Table 3B**).

**Table 3B:** Significant differences in the relative abundance of fungal species (%) ± standard error between potting soil with and without 2% chitin (*n* = 5) and their possible functions reported in literature.

Phylum	Family	Genus	Treatment		Increase or decrease	Functions (reference)
			Control	2% chitin		
Ascomycota	**Cordycipitaceae**	***Lecanicillium***	0.09 ± 0.05	1.85 ± 0.33	**20x**	PGP, chitin degradation, biocontrol and induced resistance (Goettel et al., 2008; Hirano et al., 2008; Ownley et al., 2010; Van Nam et al., 2014; Nguyen et al., 2015)
	**Pseudorotiaceae**	***Pseudogymnoascus***	0.96 ± 0.30	3.46 ± 0.26	**4x**	Biocontrol (Tagawa et al., 2010)
	Pseudorotiaceae	*Pseudeurotium*	81 ± 0.42	0.12 ± 0.02	0.07x	/
Zygomycota	**Mortierellaceae**	***Mortierella***	3.21 ± 1.73	58.13 ± 2.55	**18x**	Chitin degradation (Kim et al., 2008) and biocontrol (Tagawa et al., 2010)

*PGP, Plant growth promotion. Species **in bold** mean a significant increase in the relative abundance in the chitin treatment as compared to the control treatment. Species underlined mean a significant decrease in the relative abundance in the chitin treatment as compared to the control treatment*.

**FIGURE 4:**
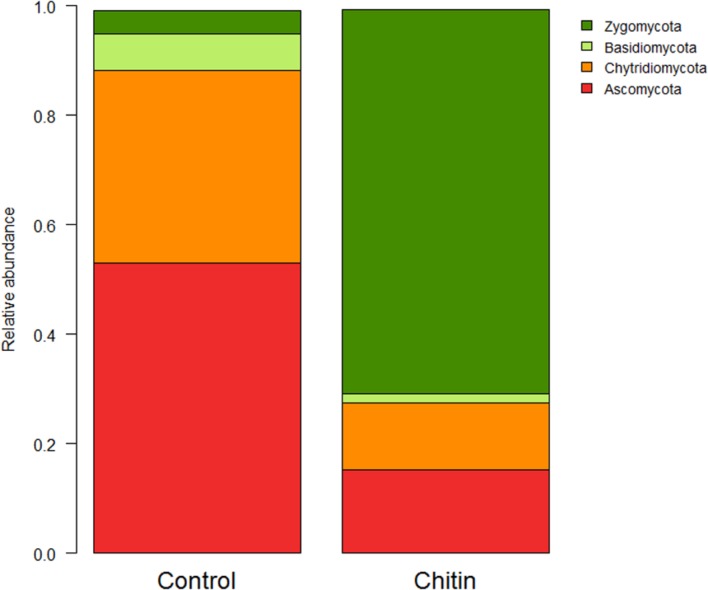
**Analysis of the fungal composition of the lettuce rhizosphere in unamended and chitin amended potting soil**. Relative abundance (percentages) of the different fungal phyla (ITS2 region) in the lettuce rhizosphere. The Cercozoa group represents only a minor part of the total fungal community (control: 0.126 ± 0.076%, chitin: 0.004 ± 0.004%) and is therefore not illustrated in the bar chart.

In total five fungal phyla were found across all samples, of which three phyla were significantly different between the two treatments: the *Ascomycota*, *Basidiomycota*, and the *Zygomycota* (*P* < 0.05, **Figure 4**). The *Zygomycota* were significantly increased in the chitin treatment, whereas the *Basidiomycota* and *Ascomycota* were significantly decreased.

**FIGURE 5:**
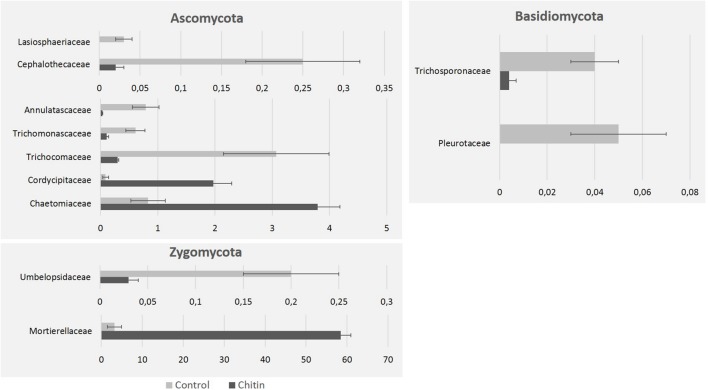
**Fungal taxonomical changes in the rhizosphere community after 2% chitin amendment to the potting soil**. The graphics represent the significant differences in relative abundance of representative families in the lettuce rhizosphere, due to the addition of chitin to the potting soil. All families belong to the phyla Zygomycota, Ascomycota, and Basidiomycota.

On family level, chitin addition altered the relative abundance of 11 fungal families significantly, in particular seven families of the *Ascomycota*, two families of the *Zygomycota* and two families of the *Basidiomycota* (**Figure 5**). Especially the *Morteriellaceae* showed an high increase, due to the genus *Morteriella* that was strongly represented and is clearly promoted by the presence of chitin in the potting soil. Two other fungal genera of the phylum *Ascomycota* increased in relative abundance due to the chitin treatment: *Lecanicillium* and *Pseudogymnoascus*. Additionally, only one genus decreased significantly in relative abundance: *Pseudeurotium*. All genera induced by the chitin included species reported in literature to be involved in biocontrol and/or chitin degradation (**Table 3B**).

## Discussion

Since farmers, consumers, and policy makers have become more aware of the impact of the use of chemical pesticides and fertilizers on human health and the environment, there is a renewed interest in the use of organic soil amendments to improve crop yield and plant resilience. It has been shown that the use of these soil amendments can have a positive influence on plant growth and development and on the suppression of plant diseases (e.g., Akhtar and Malik, 2000; Noble and Coventry, 2005; Postma and Schilder, 2015). Several studies have linked these beneficial effects to the influence of the soil amendment on the microbiome of the soil and rhizosphere of the plant. The addition of chitin for example increases the abundance of PGPR and PGPF in the soil and/or rhizosphere of the plant (Sarathchandra et al., 1996; Radwan et al., 2012; Cretoiu et al., 2013). Although chitin addition seems to control soil-borne pathogens and to enhance plant disease resistance, it was not known whether it also has an effect on the survival of human pathogens on the plant. Especially leafy vegetables are considered high risk food as they can carry human pathogens such as *E. coli* O157:H7 and *S. enterica* on their leaves (Van der Linden et al., 2013). In addition, it has been shown that biotic and abiotic stress have great influence on both the rhizosphere and phyllosphere microbiomes of lettuce (Williams et al., 2013; Williams and Marco, 2014; Erlacher et al., 2015). In the current study, we used lettuce plants grown in peat based-potting soil with and without chitin. We assessed the effect of chitin addition on (1) lettuce growth, (2) the survival of zoonotic pathogens on the lettuce leaves, and (3) rhizosphere microbial community.

Chitin addition to the soil significantly increased the fresh weight of the lettuce leaves by approximately 20%. This is in accordance to the study of Muymas et al. (2015). Chitin addition also significantly reduced the survival of *S. enterica* on the leaves. Although, not significantly in both independent experiments, also the survival of *E. coli* O157:H7 seemed to be negatively affected by the chitin amendment. The chitin soil amendment increased the absolute and relative abundance of several fungal and bacterial groups involved in plant growth promotion and in biological control. Since the roots are in direct contact with the soil, it is not surprising that a soil amendment such as chitin addition has an effect on the rhizosphere microbiome. It is less obvious, however, that it also has an effect on the survival of *Salmonella* on the leaves. It remains unclear what the exact mechanism is behind the decreased survival of *S. enterica* on the lettuce leaves. A range of beneficial agronomical responses can occur when chitin is added to the growing medium of plants (Sharp, 2013): (1) direct antibiosis against pests and pathogens of crops; (2) enhancement of beneficial microbes, both in plant defense and growth; (3) direct stimulation of plant defense responses against biotic stress; and (4) up-regulation of plant growth, development, nutrition and tolerance to abiotic stress. The three latter responses may explain our observed plant growth promotion effect and the reduced survival of *S. enterica* on the leaves, but only (2) has been measured in the current study using PLFA and amplicon sequencing. So, more research is needed to fully explain our observations. For example, next to an indirect effect via the rhizosphere microbiome, chitin can also act as a PAMP, directly triggering the immune system of the plant (de Jonge et al., 2010) and this may also explain the reduced colonization of *S. enterica* on the lettuce leaves. In addition, we also need to investigate which of the identified PGPR and PGPF can be responsible for the observed effects and what the underlying mechanism is. In accordance, a recent study showed that a PGPR bacterium (*Bacillus subtilis* UD1022) applied to the roots was able to influence the survival of human pathogens (*Listeria* and *Salmonella*) on leafy greens. This was correlated with an induction of the stomata closure by the *Bacillus* strain (Markland et al., 2015). *Bacillus subtilis* well-known PGPR effect is at least partly based on the production of surfactines, which induce plant immune system in a priming-like manner (Cawoy et al., 2014). In our study, no increase in the relative abundance of *Bacillus* species was seen, but other PGPR and PGPF were more than 10-fold increased after chitin addition, including bacterial species belonging the genera *Cellvibrio*, *Pedobacter*, *Dyadobacter*, and *Streptomyces* and fungal species belonging to the genera *Lecanicillium* and *Mortierella.* This confirms previous observations of De Boer et al. (1999), who showed that the rapid degradation of chitin in dune soils was most likely due to fast-growing *Mortierella* sp., whereas *Streptomyces* sp. and slow-growing fungal species (such as *Verticillium* sp, now partially re-classified as *Lecanicillium* sp.) were shown to be more involved in the degradation of chitin after prolonged incubation.

Our study addresses some limitations of previous studies and extends our knowledge about the effect of chitin on below ground microbiology because (1) rhizosphere samples were studied instead of bulk field soil; (2) both the fungal and bacterial community were assessed using Illumina sequencing; and (3) PLFA was used as an additional technique which allows quantification of microbial biomass. In our study, incorporating chitin in peat-based potting soil for almost 2 months significantly increased the relative abundance of the *Proteobacteria*, *Bacteroidetes*, and *Actinobacteria* in the rhizosphere, while those of the *Verrucomicrobia* and the *Acidobacteria* were significantly decreased. This confirms previous results that describe an increase in the relative abundance of *Proteobacteria* (Jacquiod et al., 2013; Cretoiu et al., 2014), *Actinobacteria* (Jacquiod et al., 2013), and *Bacteroidetes* (Cretoiu et al., 2014) due to chitin amendment in field soil. PLFA analyses showed a twofold increase in both fungal and bacterial biomass in the rhizosphere due to chitin amendment. Cretoiu et al. (2013) however, showed a 10-fold increase in bacterial abundance, but a 10-fold decrease in fungal abundance in chitin-amended field soil compared to unamended field soil using qPCR. These comparisons show that the main trends at group or phylum level are similar, even though the experimental set-up differed (e.g., field soil vs. potting soil, soil vs. rhizosphere sampling). Based on our results and others, chitin addition thus gives reproducible shifts in microbial community even in very different soil systems. At lower taxonomic levels, differences are more common due to the specific niche of the rhizosphere, which is expected to be different from bulk soil (e.g., Lundberg et al., 2012; Peiffer et al., 2013). To the best of our knowledge, the presented study is the first study to use amplicon sequencing of the fungal ITS2 region to assess the effect of chitin soil amendment on the rhizosphere microbiome. We showed that addition of chitin to soil influenced the fungal composition of the rhizosphere, in which three major phyla shifted: an increase in the *Zygomycota* and a decrease in the relative abundance of the *Ascomycota* and *Basidiomycota.* We showed that the relative abundance of the fungal genera *Lecanicillium* and *Mortierella* was highly increased, both containing species involved in plant growth promotion, chitin degradation and biological control. Additionally, *Mortierella* sp. belonging to a the complex group of the *Mortierellales* (Wagner et al., 2013) might play an important component in the phosphorus cycling of the plant (Curlevski et al., 2010).

Both PLFA and amplicon sequencing was used for studying the rhizobiome of the lettuce plants. For both techniques: (1) different soil sampling was done and (2) different information was gathered. First, for the amplicon sequencing, 250 mg rhizosphere soil was taken as defined by Lundberg et al. (2012). Because of the amount of soil needed for PLFA analysis (6 g), it is impossible to do same soil sampling as for the amplicon sequencing. For this technique, 6 g of soil was taken from the pots. These pots were fully colonized by the lettuce roots (**Supplementary Figure S2**), so soil very close to the roots was taken and we believe that this can still be defined as rhizosphere soil. However, this is a very particular concept of rhizosphere, being very artificial. Due to this experimental restriction, the high root density could be very different from a natural situation, not only about the access of the chitin, but also about the microbiome present. Second, amplicon sequencing is known to give reliable information on microbial taxonomy, especially for higher order identification (Poretsky et al., 2014). Also information on species richness and diversity can be calculated. However, using the amplicon sequencing technique, the relative abundances are calculated and we do not have information on the real microbial biomass. PLFA analysis on the other hand provides complementary data on the total biomass and the biomass per microbial group. To make a comparison between the two techniques possible, the relative abundances of the PLFA biomarkers was also calculated, showing an increase in the relative abundance of the Gram negative-bacteria, similar as shown with the amplicon sequencing. This could not be confirmed for the Gram-positive bacteria, showing a decrease for PLFA analysis and no significant effect for the 16S rDNA amplicon sequencing.

## Conclusion

In the current study, we demonstrated that the chitin soil amendment strategy which was previously known to be effective against plant pathogens (e.g., Dutta and Isaac, 1979; Hallmann et al., 1999; Cretoiu et al., 2013) also is able to control *Salmonella* on leafy greens. Chitin amendment in potting soil increased lettuce growth and had a decreasing effect on the survival of human pathogens on the leaves. These two effects were accompanied with changes in the rhizosphere microbiome. The observations that chitin soil amendment can increase plant yield, including lettuce, and can change soil and rhizosphere microbiology are not new, and our study confirms the results seen by other studies (e.g., Sarathchandra et al., 1996; Radwan et al., 2012; Cretoiu et al., 2013, 2014; Jacquiod et al., 2013; Muymas et al., 2015). This is, however, the first study to show that chitin soil amendment can have an effect on the survival of human pathogens on leafy vegetables. This addresses some of the knowledge gaps between food safety and plant sciences and similar studies combining both research fields are expected (Markland and Kniel, 2015; Melotto et al., 2015).

## Author Contributions

JD, SS, IVdL, BC, MH, and MM were involved in the design and the supervision of the experiments. JD wrote the first draft and finalized the manuscript. SS and CVM conducted the plant experiments and the bacterial counting. CDT and AH conducted the amplicon sequencing, the bio-informatics and statistical analysis of the NGS and PLFA data. All authors contributed to the writing of the manuscript and approved submission.

## Conflict of Interest Statement

The authors declare that the research was conducted in the absence of any commercial or financial relationships that could be construed as a potential conflict of interest.

## Supplementary material

FIGURE S1 **Two lettuce plants after 55 days of growth in the growth chamber (left = control, right = chitin treatment)**.

FIGURE S2 **Root development of two lettuce plants after 55 days of growth in the growth chamber (left = control, right = chitin treatment)**.

FIGURE S3 ***Escherichia coli* O157:H7 MB3885 dynamics on middle-aged lettuce leaves at 0, 4, and 8 days after spray inoculation analyzed by plating as described by Van der Linden et al. (2013)**. Full lines represent control plants, while dashed lines represent chitin treated plants. The data are calculated from the log-transformed values of the pathogen per gram tissue from two independent experiments (*n* = 2 plants or 6 leaves for day 0 and *n* = 6 plants or 18 leaves for day 4 and 8). Asterisk means significantly different between the chitin and the control treatment. Bars represent standard errors.

FIGURE S4 **Principal coordinate analysis (PCoA) of Bray–Curtis dissimilarity matrix calculated from the phospholipid fatty acids of chitin amended and unamended potting soil (= control) at the 55 days after planting**. First PCoA axis represents 94.9% of the variability of the dataset, second axis 2.5%.

FIGURE S5 **Rarefaction curve of the 16S V3–V4 sequencing data for the rhizosphere of lettuce grown in unamended (= control) and chitin amended (= chitin) potting soil**. Shown are the mean rarefaction curve for each treatment (*n* = 5) with standard error margins. Rarefaction depth for this study was set at 50,000 sequences as convergence seems to be reached for both treatments.

FIGURE S6 **Principal coordinate analysis profile of pairwise community dissimilarity (Bray–Curtis) indices of 16S sequencing data of the lettuce rhizosphere grown in chitin amended (yellow) and unamended (brown) potting soil**. First and second axes represent 51.8 and 17.6% of the variance in the dataset respectively.

FIGURE S7 **Rarefaction curve of the ITS2 sequencing data for the rhizosphere of lettuce grown in unamended and chitin amended potting soil**. Shown are the mean rarefaction curve for each treatment (*n* = 5) with standard error margins. Rarefaction depth for this study was set at 10,000 sequences as convergence seems to be reached for both treatments.

FIGURE S8 **Principal coordinate analysis profile of pairwise community dissimilarity (Bray–Curtis) indices of the ITS2 sequencing data of the lettuce rhizosphere grown in chitin amended (yellow) and unamended (brown) potting soil**. First and second axes represent 64.8 and 18.8% of the variance in the dataset respectively.
