# Supplementary figures and images for: Chitin Mixed in Potting Soil Alters Lettuce Growth, the Survival of Zoonotic Bacteria on the Leaves and Associated Rhizosphere Microbiology
Source: Front Microbiol. 2016 Apr 21;7:565. doi: 10.3389/fmicb.2016.00565 (PMC4838818; doi:10.3389/fmicb.2016.00565)

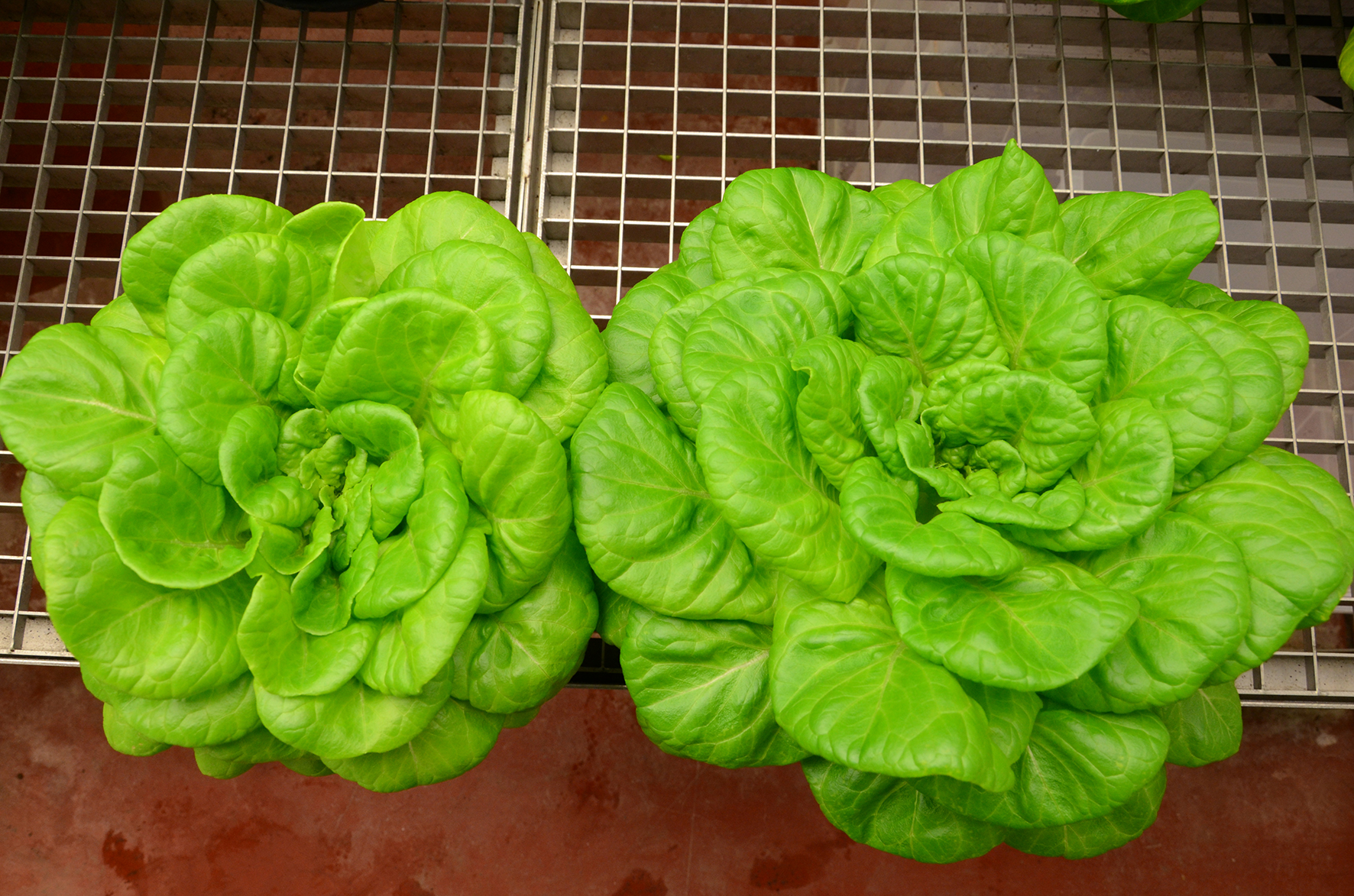

Supplement: FIGURE S1 — Two lettuce plants after 55 days of growth in the growth chamber (left = control, right = chitin treatment). [file Image_1.JPG]

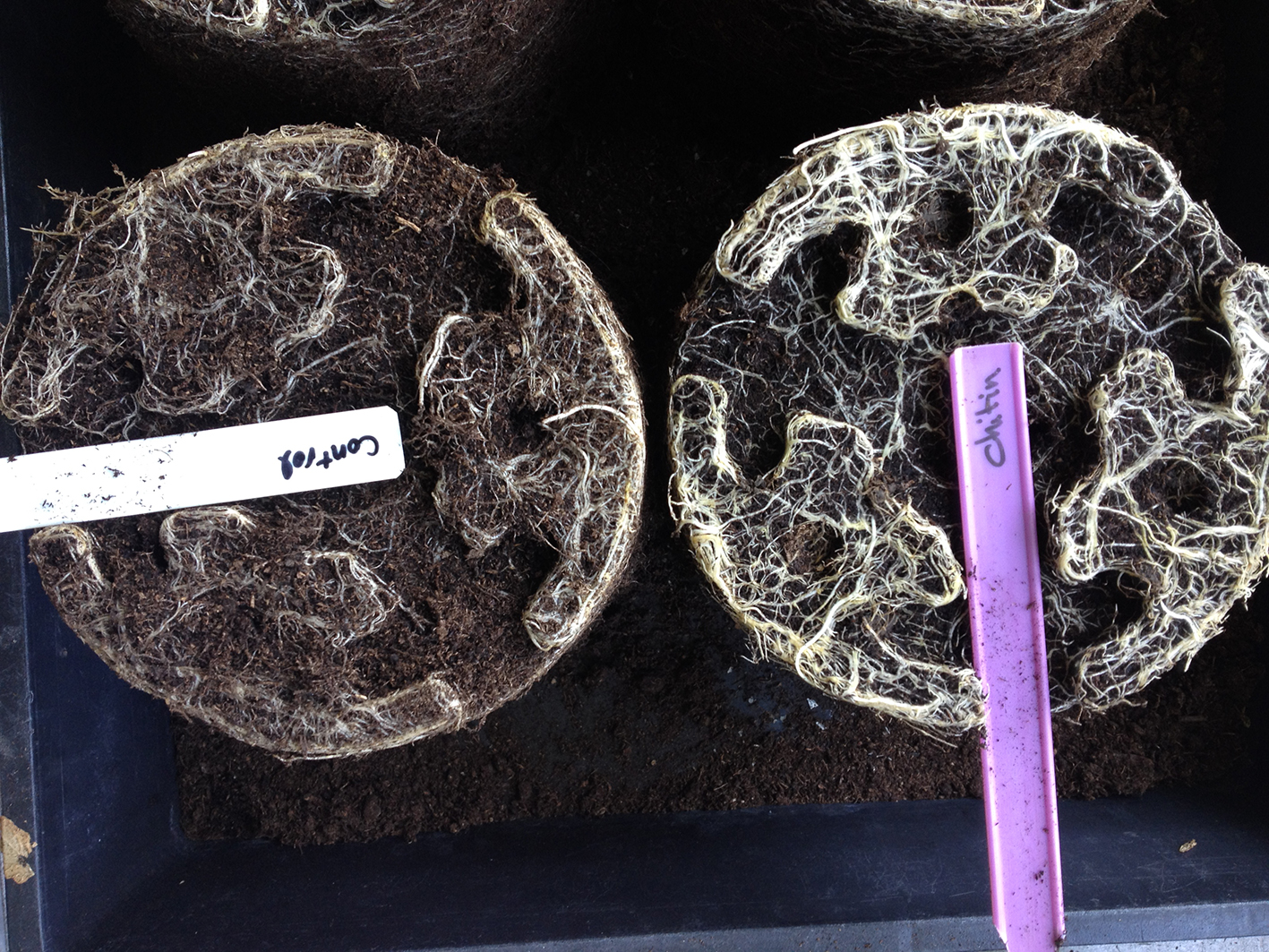

Supplement: FIGURE S2 — Root development of two lettuce plants after 55 days of growth in the growth chamber (left = control, right = chitin treatment). [file Image_2.JPG]

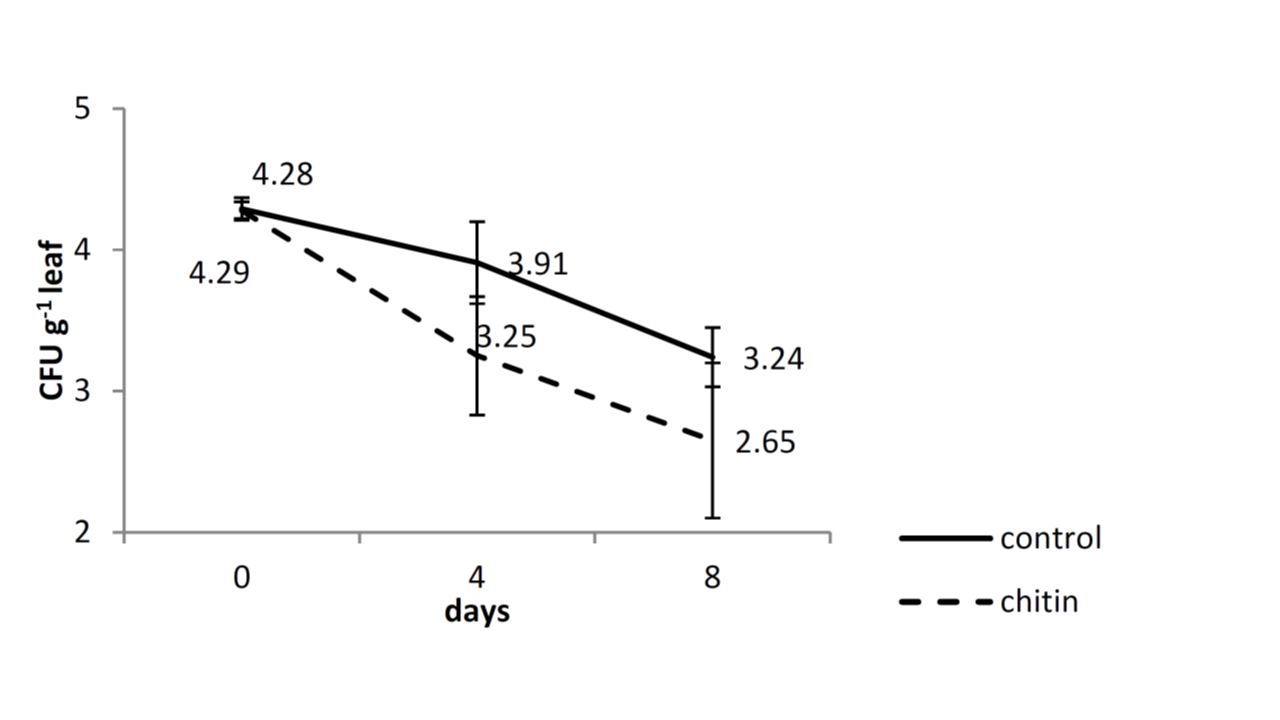

Supplement: FIGURE S3 — Escherichia coli O157:H7 MB3885 dynamics on middle-aged lettuce leaves at 0, 4, and 8 days after spray inoculation analyzed by plating as described by Van der Linden et al. (2013). Full lines represent control plants, while dashed lines represent chitin treated plants. The data are calculated from the log-transformed values of the pathogen per gram tissue from two independent experiments (n = 2 plants or 6 leaves for day 0 and n = 6 plants or 18 leaves for day 4 and 8). Asterisk means significantly different between the chitin and the control treatment. Bars represent standard errors. [file Image_3.TIF]

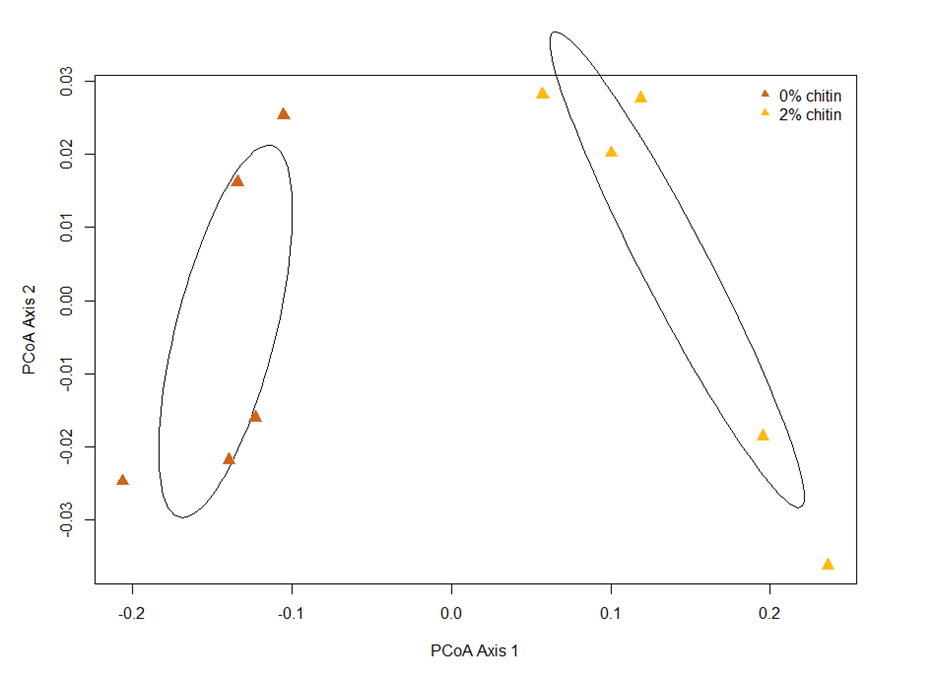

Supplement: FIGURE S4 — Principal coordinate analysis (PCoA) of Bray–Curtis dissimilarity matrix calculated from the phospholipid fatty acids of chitin amended and unamended potting soil (= control) at the 55 days after planting. First PCoA axis represents 94.9% of the variability of the dataset, second axis 2.5%. [file Image_4.TIF]

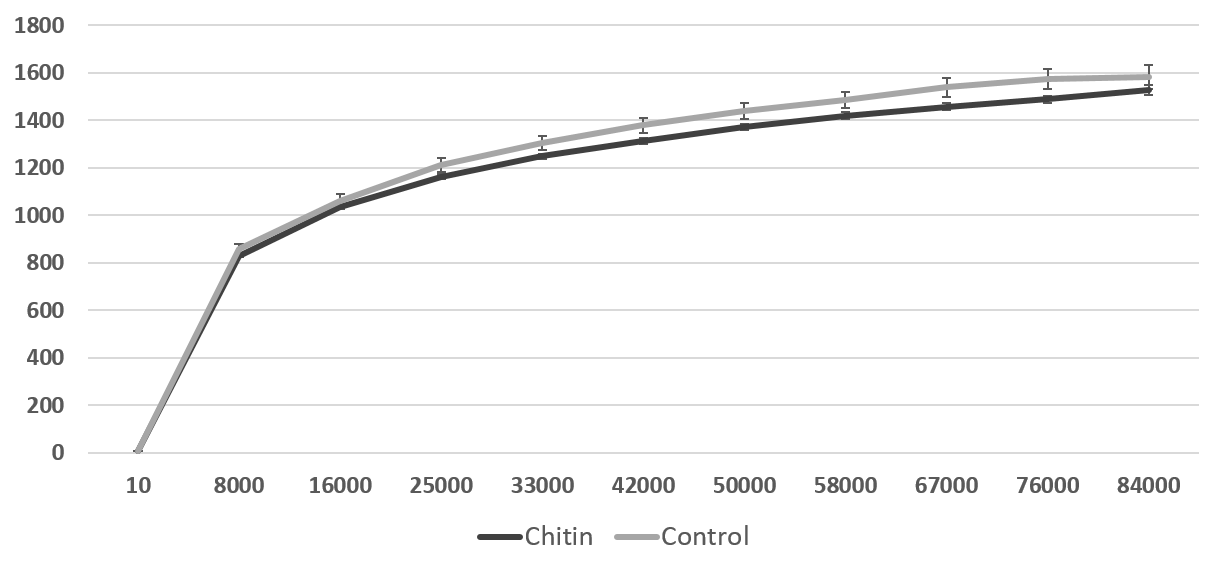

Supplement: FIGURE S5 — Rarefaction curve of the 16S V3–V4 sequencing data for the rhizosphere of lettuce grown in unamended (= control) and chitin amended (= chitin) potting soil. Shown are the mean rarefaction curve for each treatment (n = 5) with standard error margins. Rarefaction depth for this study was set at 50,000 sequences as convergence seems to be reached for both treatments. [file Image_5.TIF]

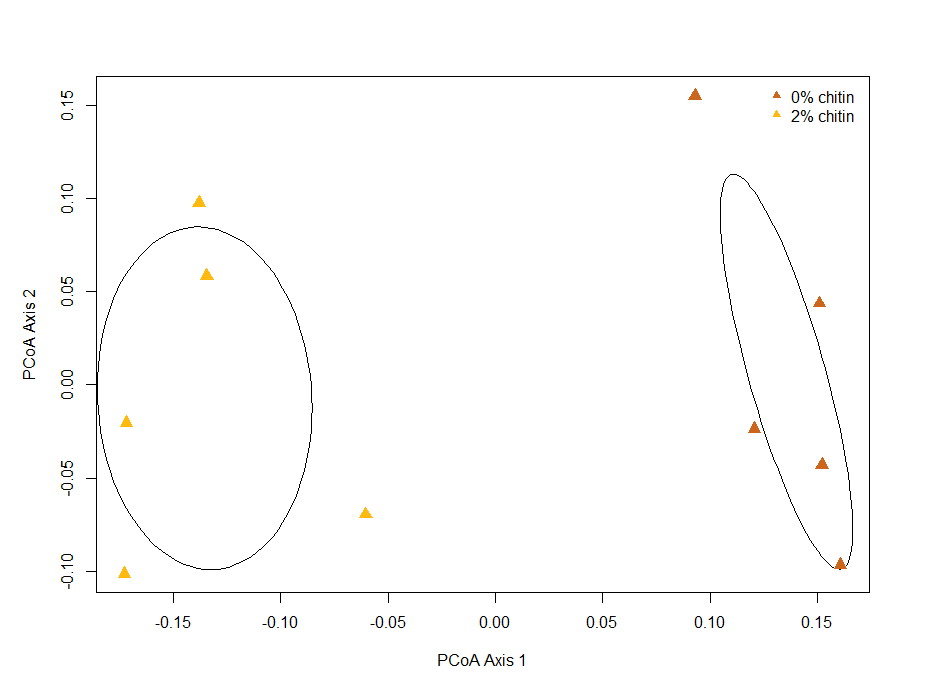

Supplement: FIGURE S6 — Principal coordinate analysis profile of pairwise community dissimilarity (Bray–Curtis) indices of 16S sequencing data of the lettuce rhizosphere grown in chitin amended (yellow) and unamended (brown) potting soil. First and second axes represent 51.8 and 17.6% of the variance in the dataset respectively. [file Image_6.TIF]

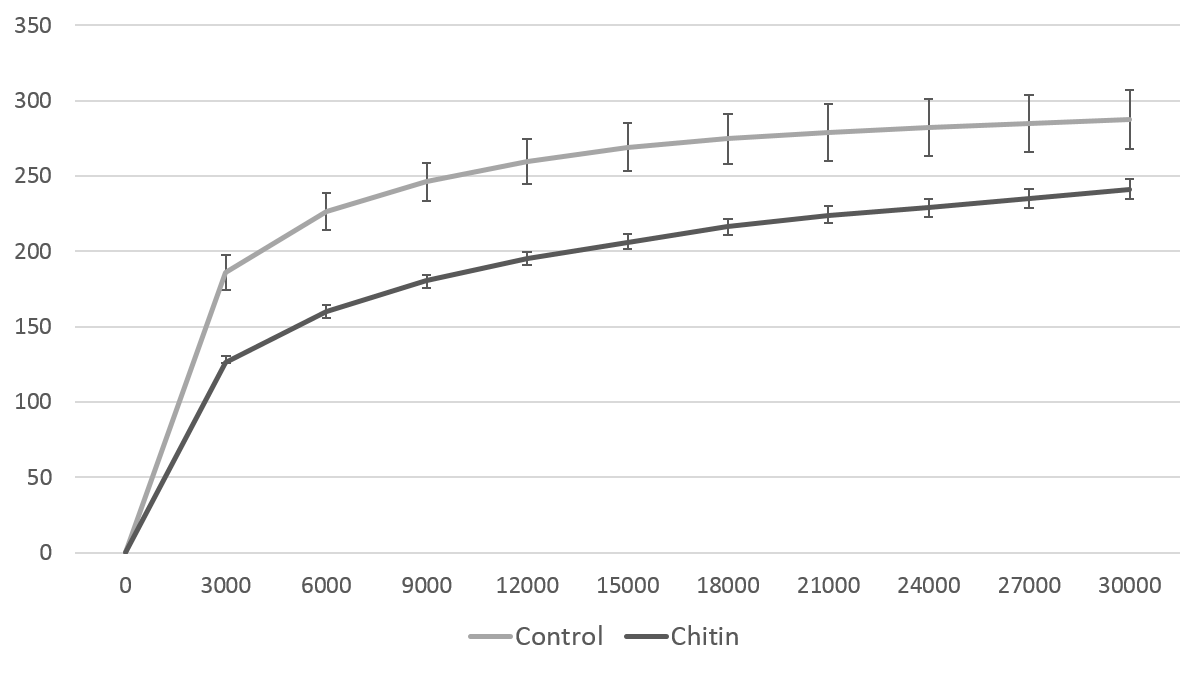

Supplement: FIGURE S7 — Rarefaction curve of the ITS2 sequencing data for the rhizosphere of lettuce grown in unamended and chitin amended potting soil. Shown are the mean rarefaction curve for each treatment (n = 5) with standard error margins. Rarefaction depth for this study was set at 10,000 sequences as convergence seems to be reached for both treatments. [file Image_7.TIF]

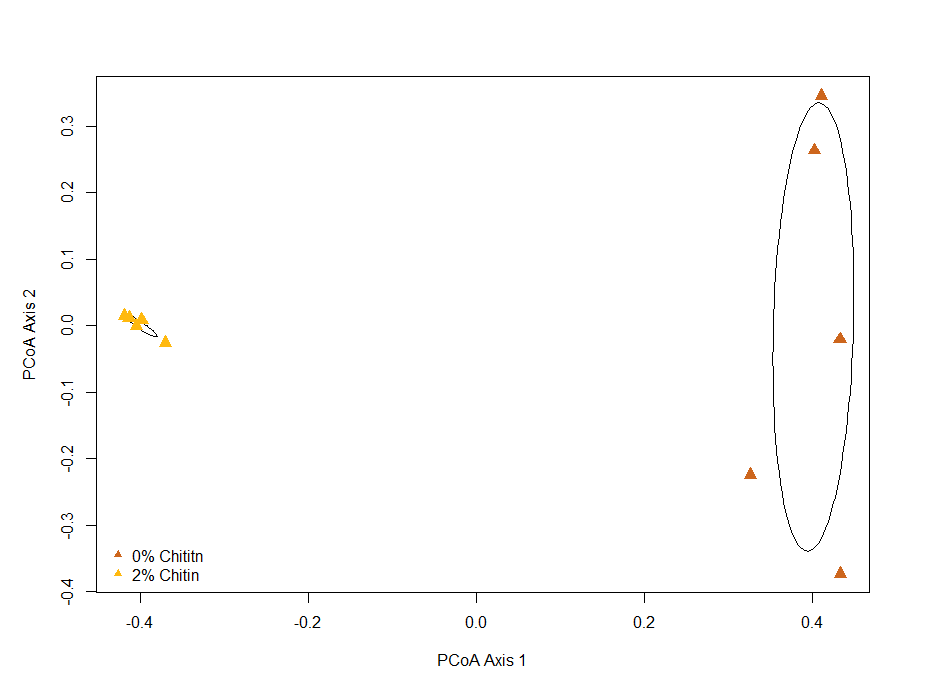

Supplement: FIGURE S8 — Principal coordinate analysis profile of pairwise community dissimilarity (Bray–Curtis) indices of the ITS2 sequencing data of the lettuce rhizosphere grown in chitin amended (yellow) and unamended (brown) potting soil. First and second axes represent 64.8 and 18.8% of the variance in the dataset respectively. [file Image_8.TIF]
